# Supplementary material for: Identification of novel human microcephaly-linked protein Mtss2 that mediates cortical progenitor cell division and corticogenesis through Nedd9-RhoA
Source: eLife. 2025 Jul 23;13:RP92748. doi: 10.7554/eLife.92748 (PMC12286603; doi:10.7554/eLife.92748)
Supplement: Figure 4—source data 1. [file elife-92748-fig4-data1.pdf]

WB: anti-Nedd9

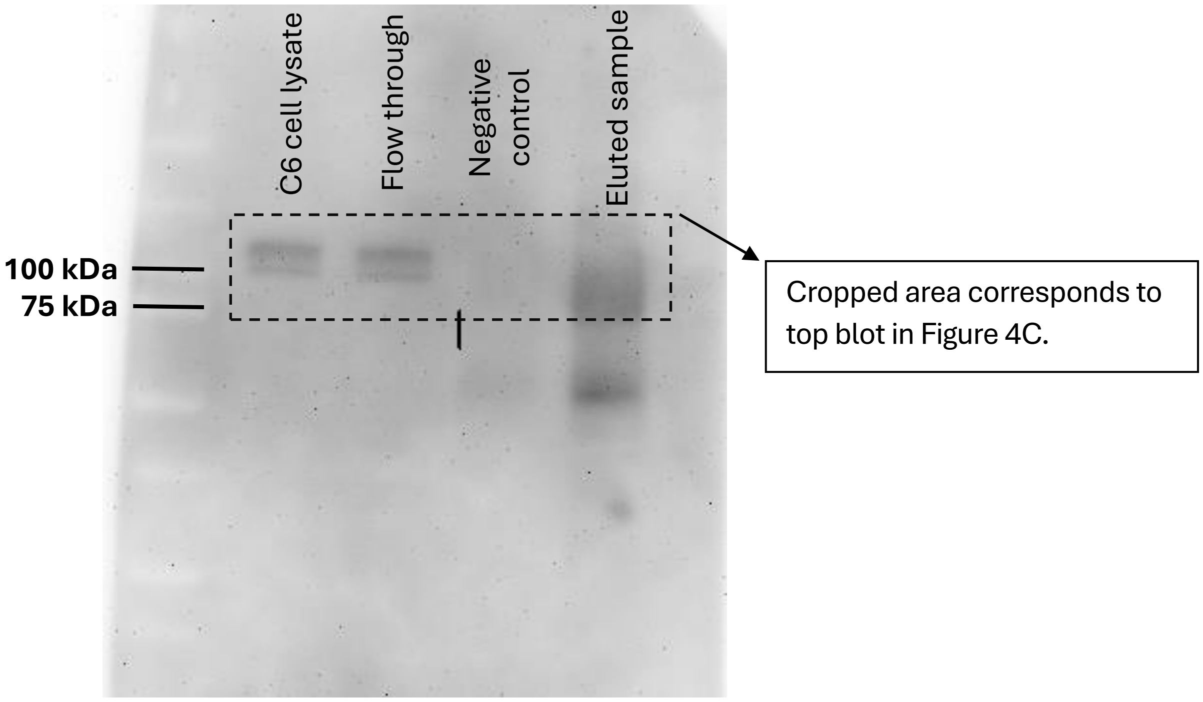

WB: anti-Abba

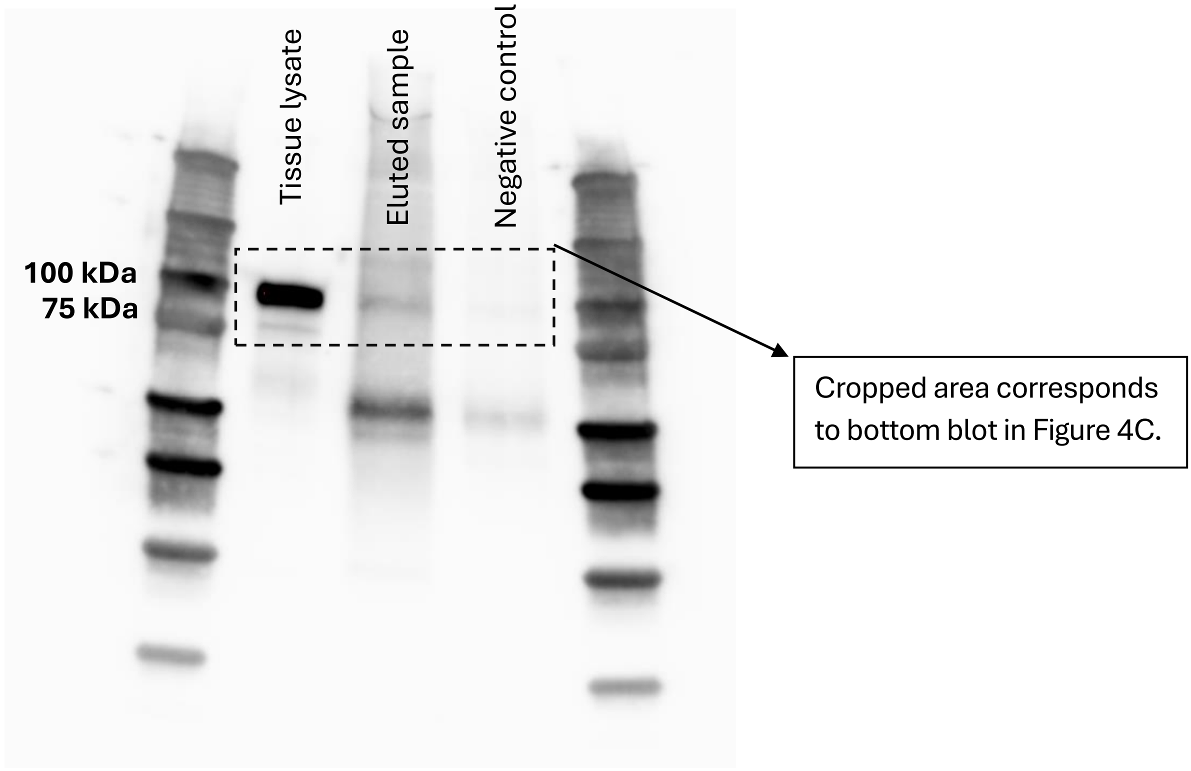

WB: anti-Abba

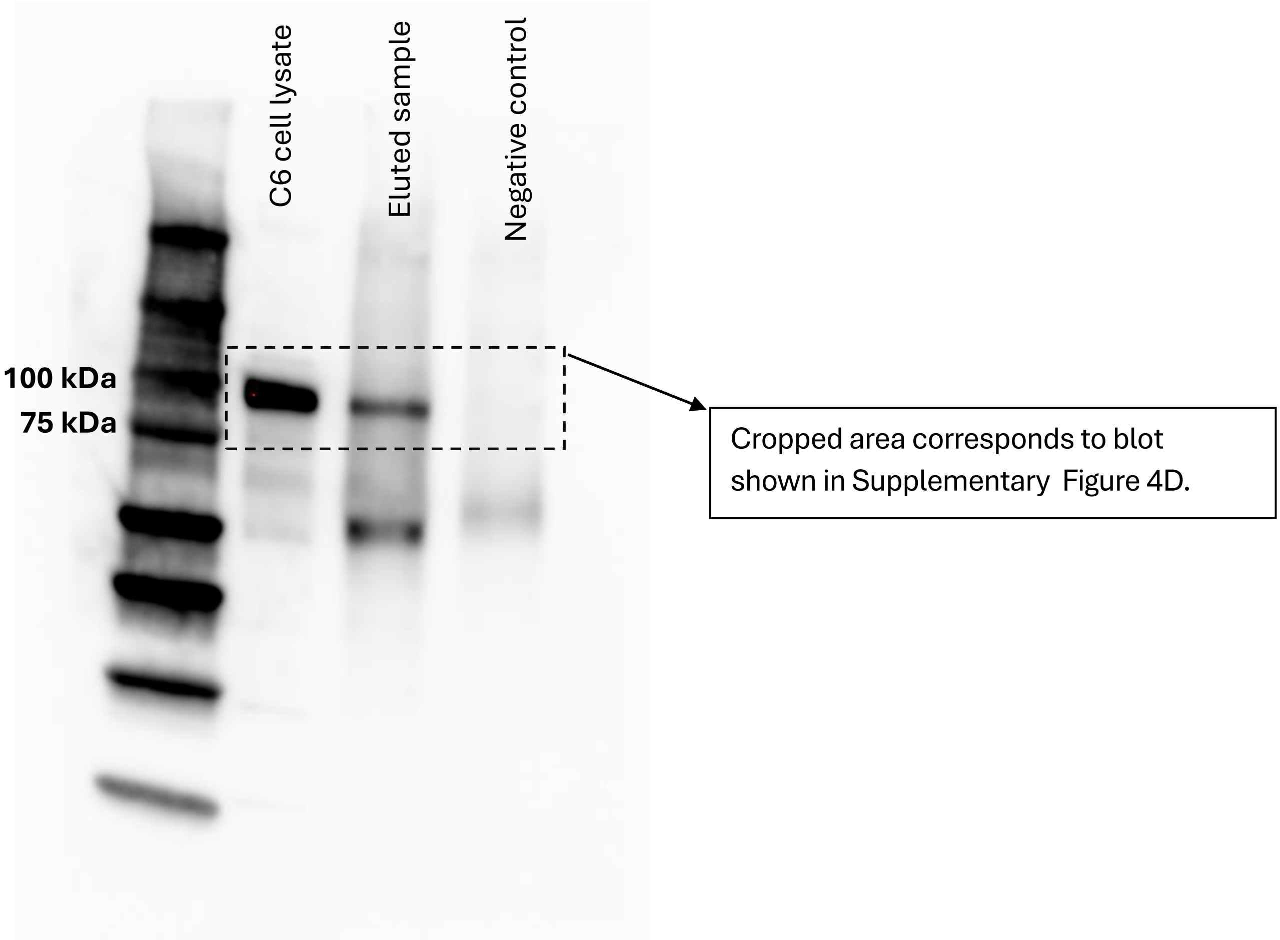
C6 cell lysate
Eluted sample
Negative control
100 kDa
75 kDa
Cropped area corresponds to blot shown in Supplementary Figure 4D.
