# Supplementary figures and images for: Identification of novel human microcephaly-linked protein Mtss2 that mediates cortical progenitor cell division and corticogenesis through Nedd9-RhoA
Source: eLife. 2025 Jul 23;13:RP92748. doi: 10.7554/eLife.92748 (PMC12286603; doi:10.7554/eLife.92748)

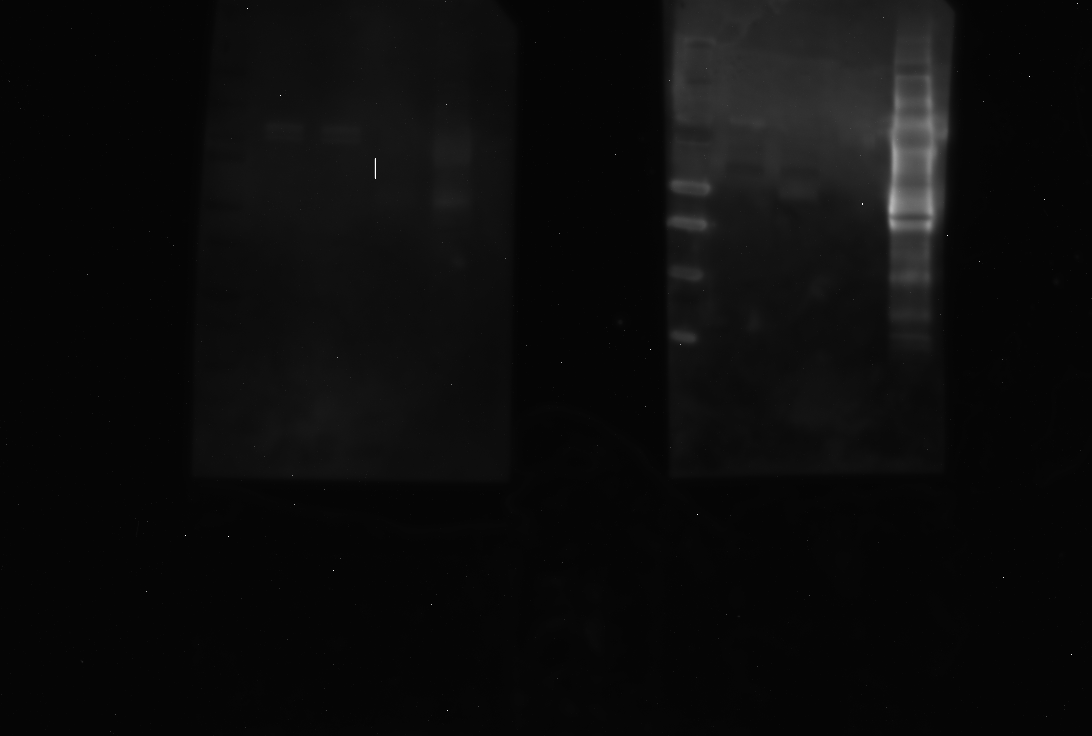

Supplement: Figure 4—source data 2. [file elife-92748-fig4-data2.zip › Figure 4 C-source data 2 -anti-Nedd9 WB/29_05_19_15min_HS_Gel1_Anti-ABBA_Anti-NEDD9.TIF]

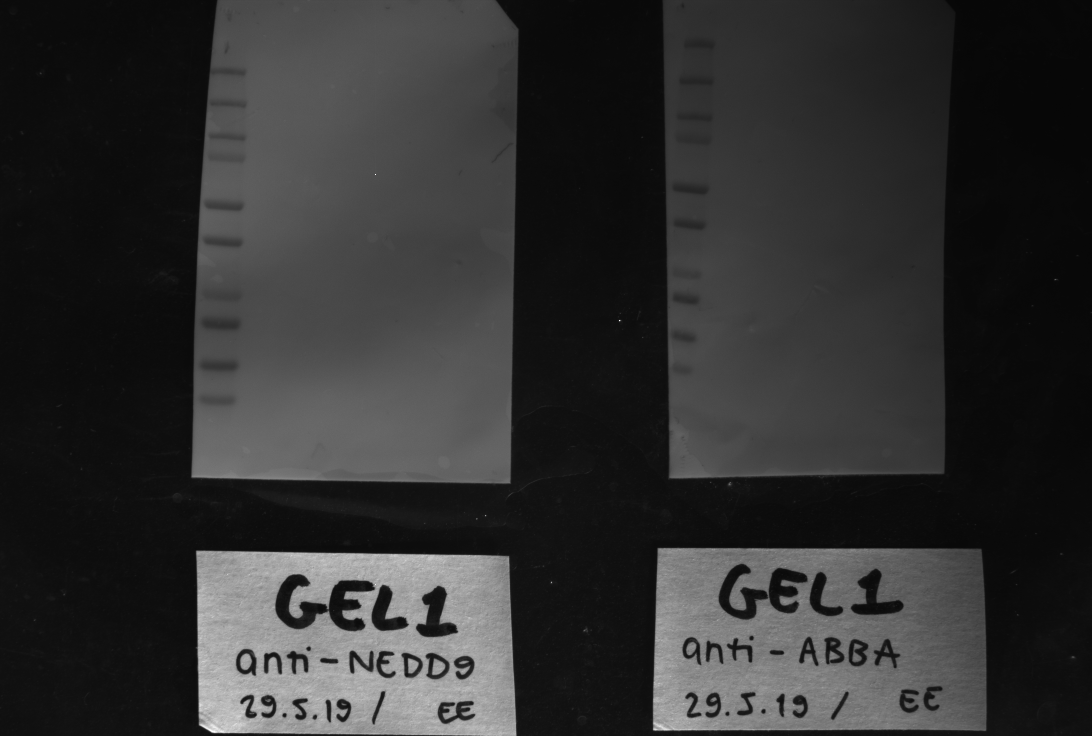

Supplement: Figure 4—source data 2. [file elife-92748-fig4-data2.zip › Figure 4 C-source data 2 -anti-Nedd9 WB/29_05_19_Bright_Gel1_Anti-ABBA_Anti-NEDD9.TIF]

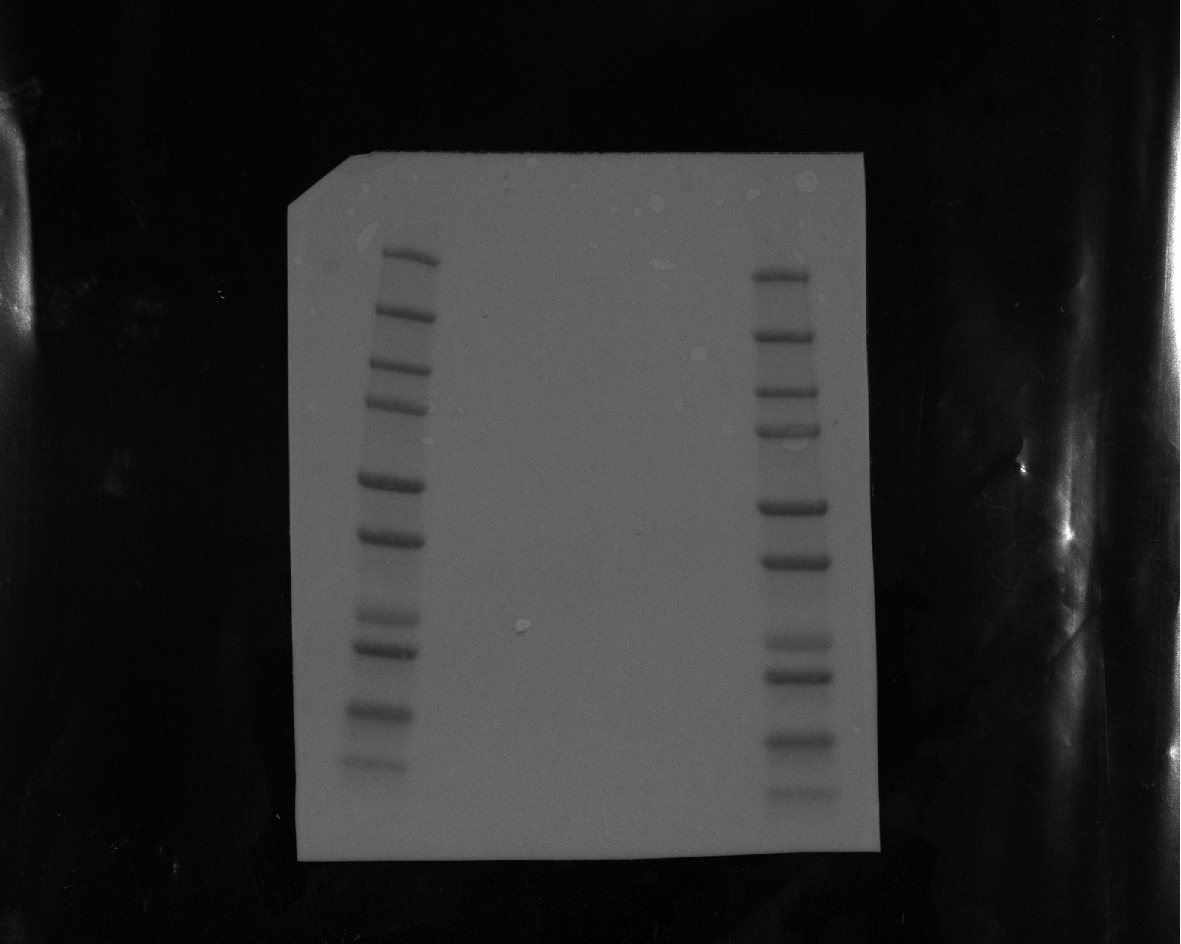

Supplement: Figure 4—source data 3. [file elife-92748-fig4-data3.zip › Figure 4 C-source data 3-anti-Abba WB/Saarikangas 2023-01-16 17h26m54s_Colorimetric.jpg]

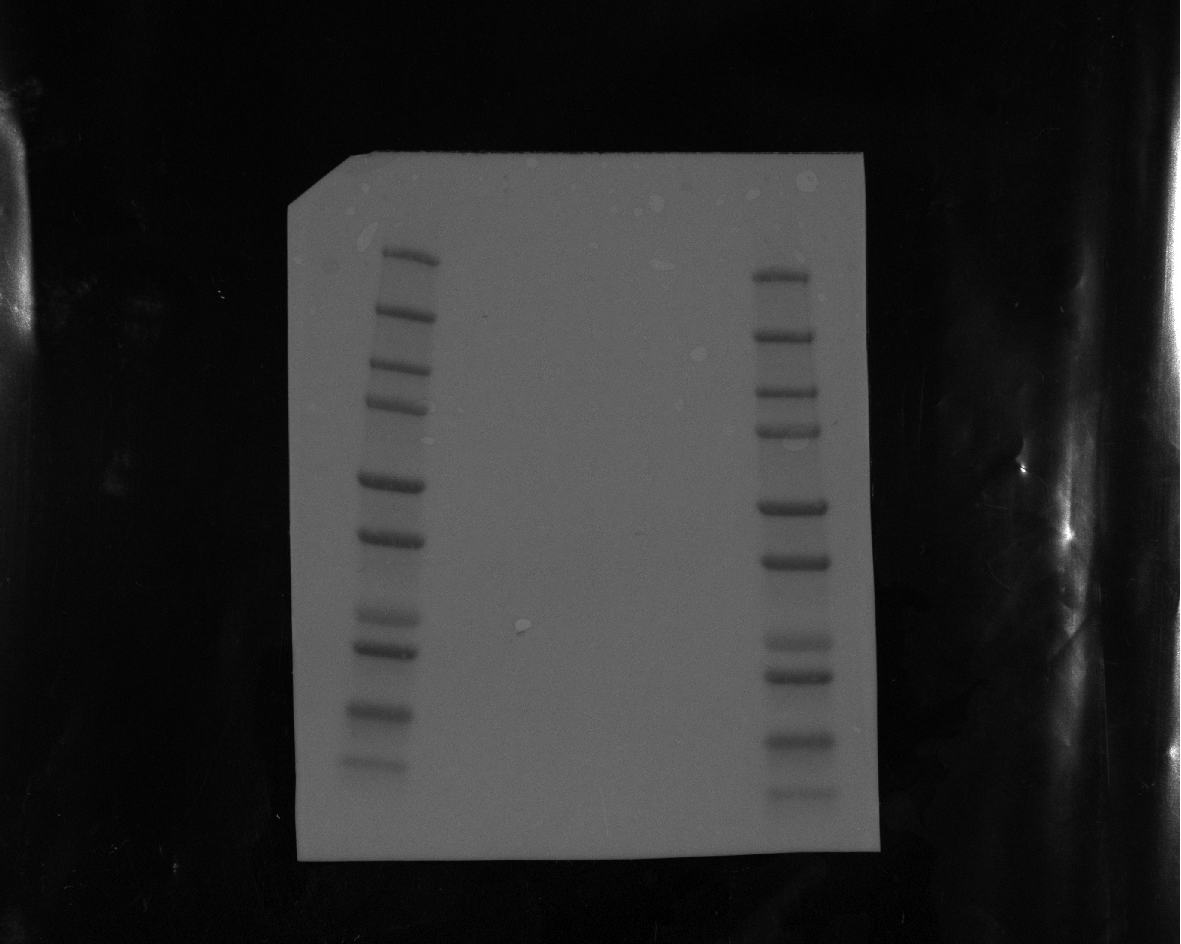

Supplement: Figure 4—source data 3. [file elife-92748-fig4-data3.zip › Figure 4 C-source data 3-anti-Abba WB/Saarikangas 2023-01-16 17h26m54s_Colorimetric.tif]

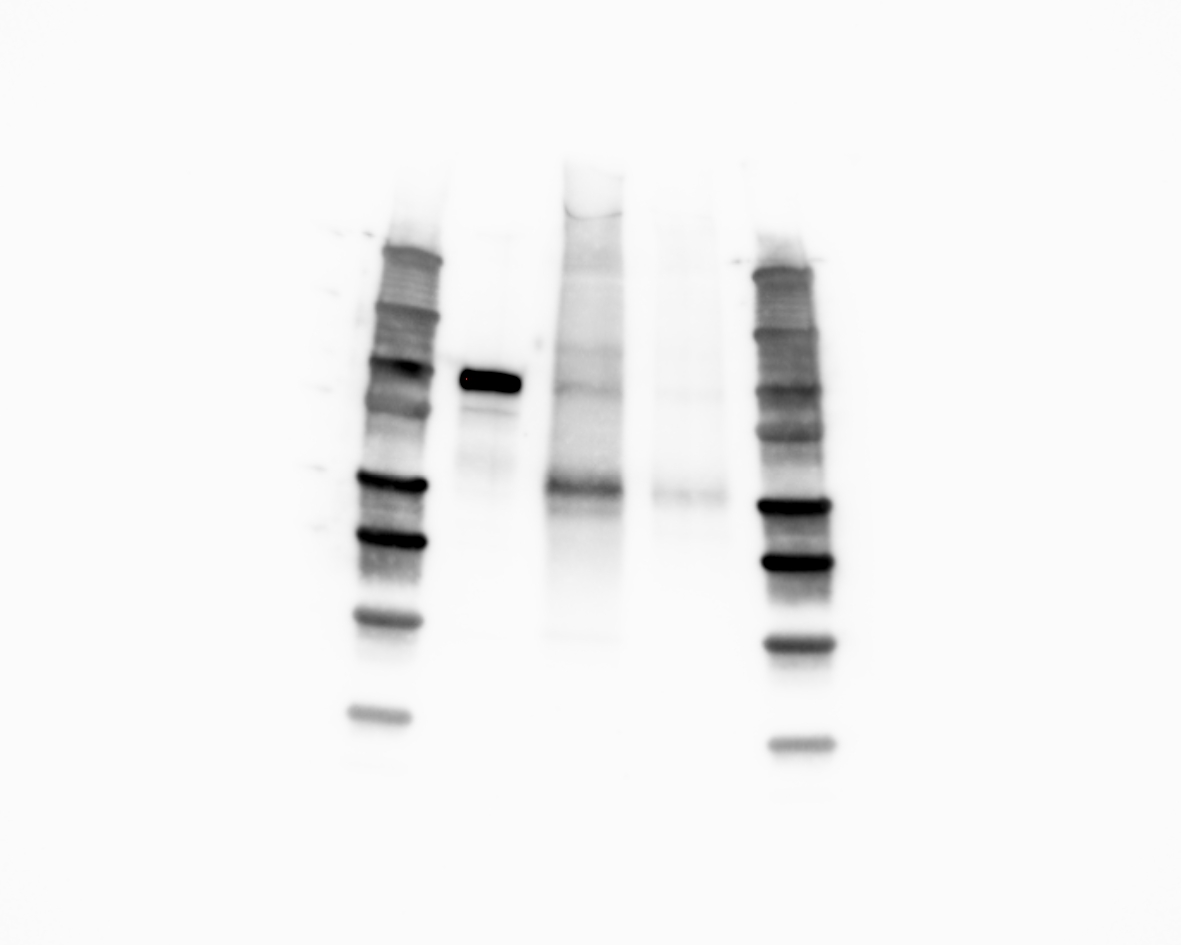

Supplement: Figure 4—source data 3. [file elife-92748-fig4-data3.zip › Figure 4 C-source data 3-anti-Abba WB/Saarikangas 2023-01-16 17h27m35s_Chemiluminescence.jpg]

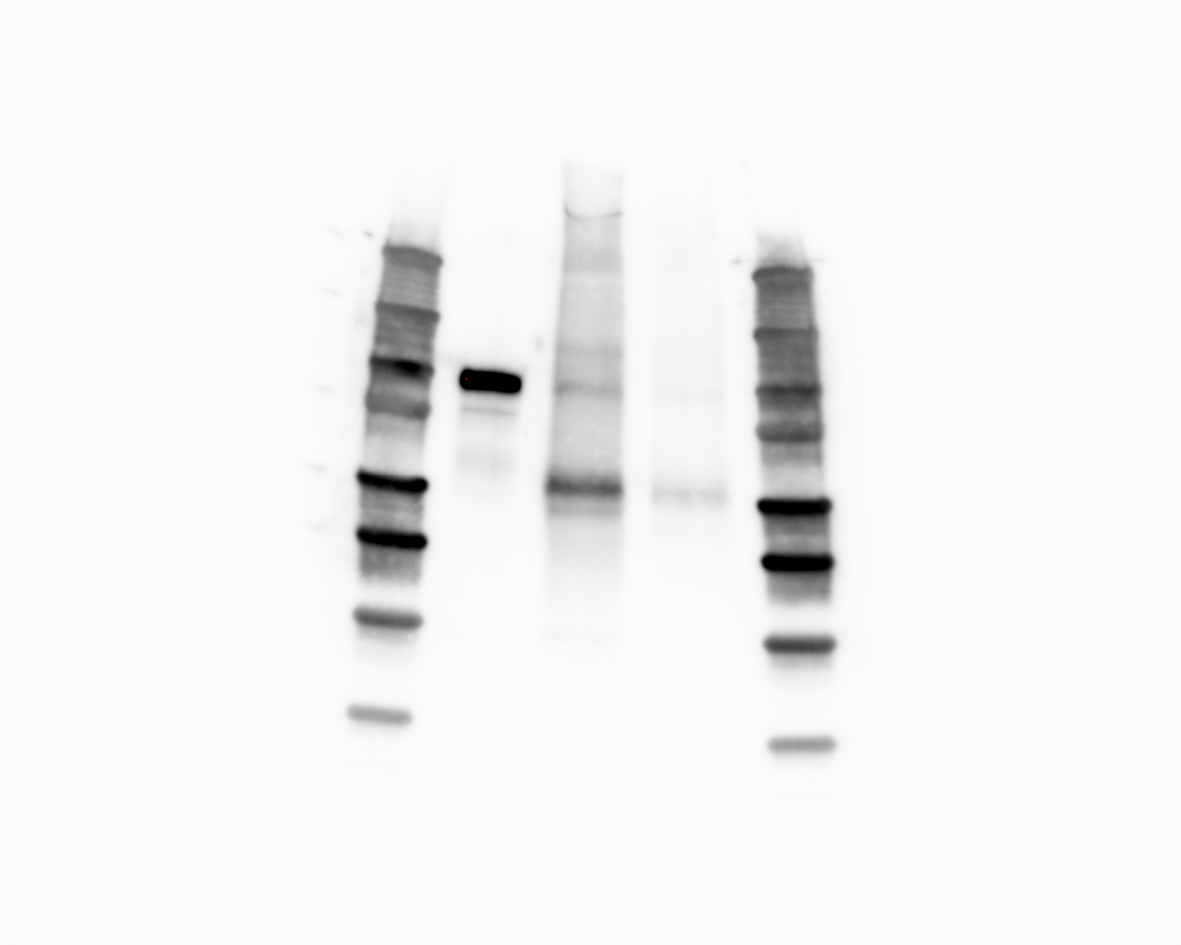

Supplement: Figure 4—source data 3. [file elife-92748-fig4-data3.zip › Figure 4 C-source data 3-anti-Abba WB/Saarikangas 2023-01-16 17h27m35s_Chemiluminescence.tif]

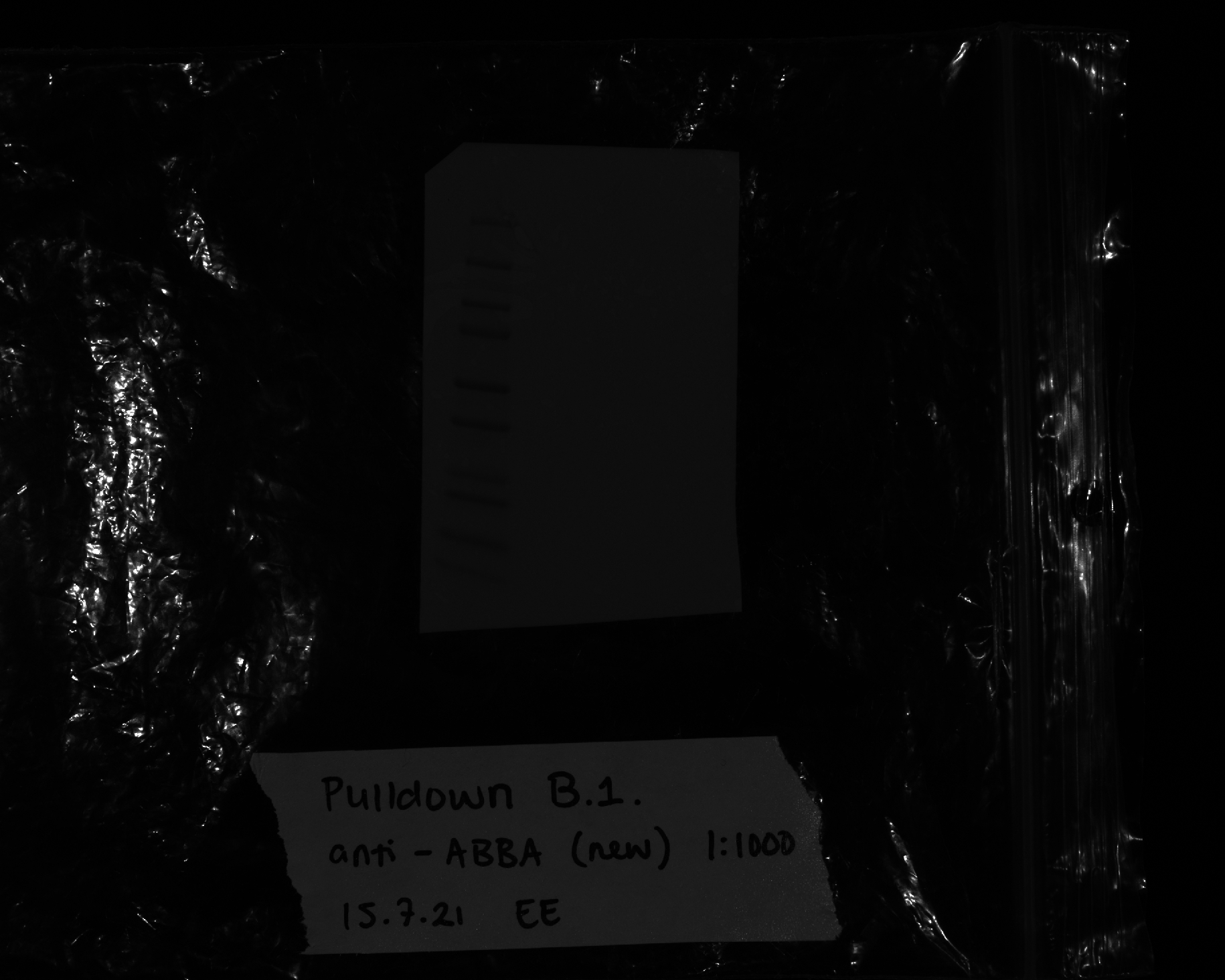

Supplement: Figure 4—figure supplement 1—source data 1. [file elife-92748-fig4-figsupp1-data1.zip › Figure 4 D-figure Supplemental-Source data-anti-Abba WB_/Saarikangas 2021-07-15 20h57m41s_Colorimetric.jpg]

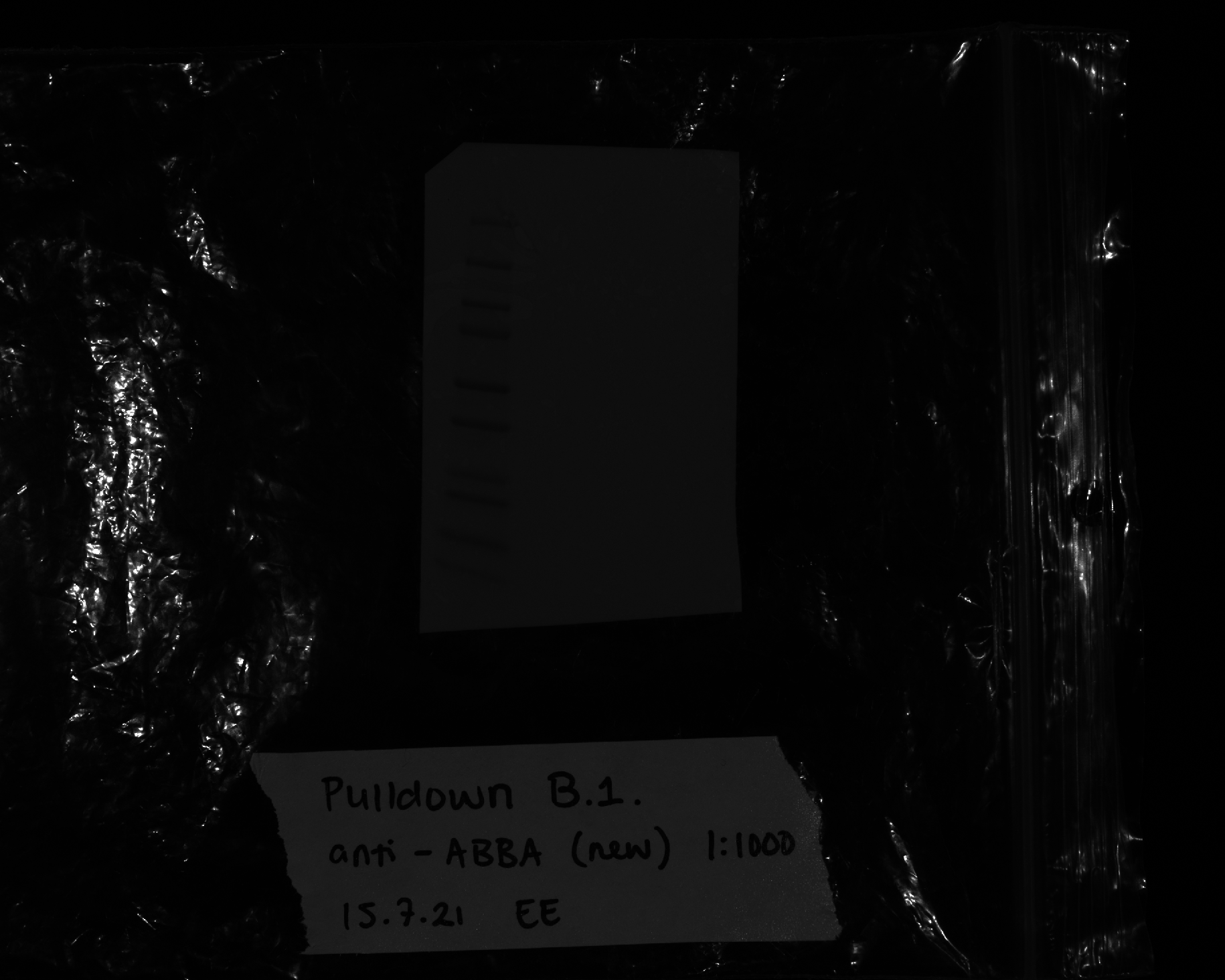

Supplement: Figure 4—figure supplement 1—source data 1. [file elife-92748-fig4-figsupp1-data1.zip › Figure 4 D-figure Supplemental-Source data-anti-Abba WB_/Saarikangas 2021-07-15 20h57m41s_Colorimetric.tif]

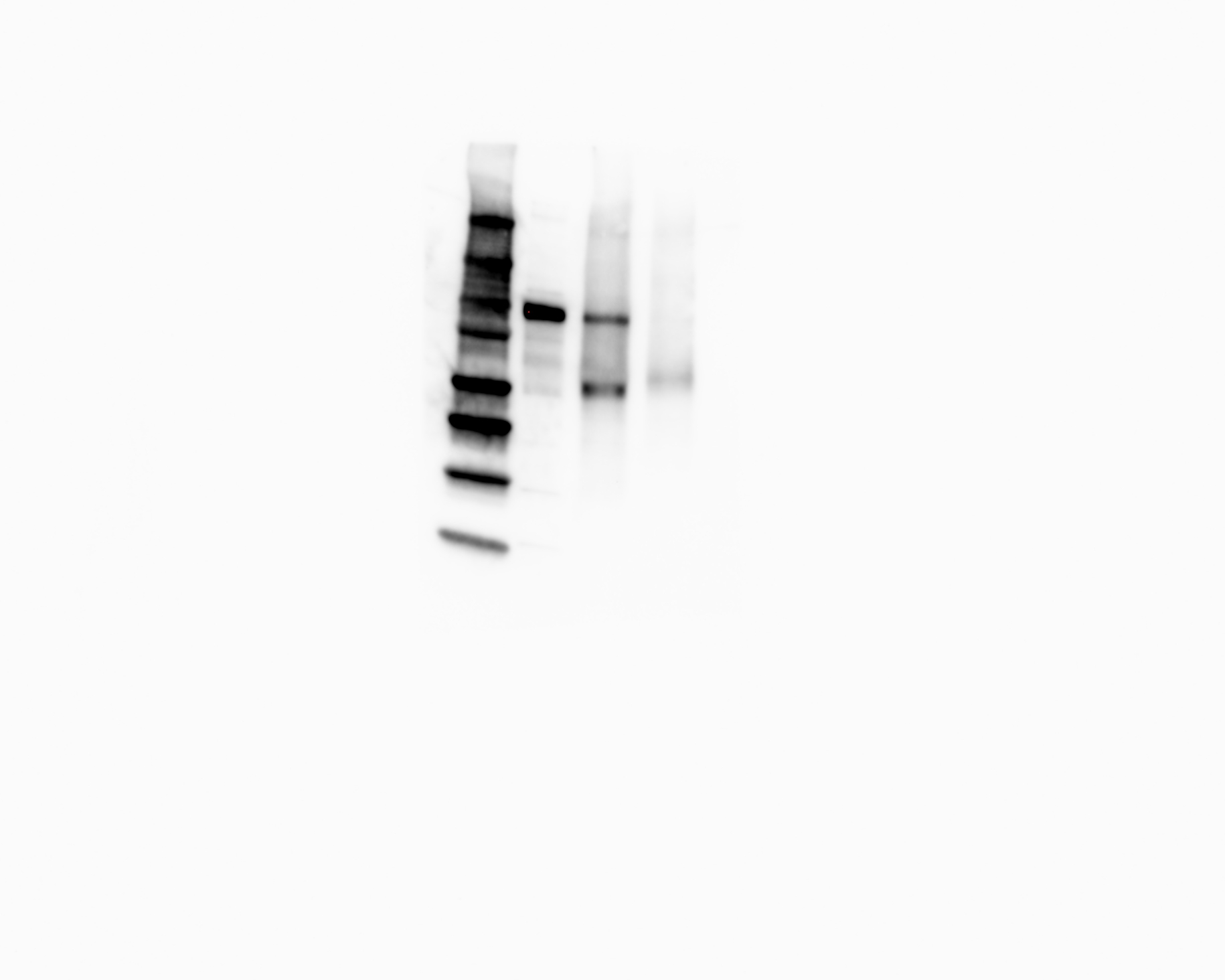

Supplement: Figure 4—figure supplement 1—source data 1. [file elife-92748-fig4-figsupp1-data1.zip › Figure 4 D-figure Supplemental-Source data-anti-Abba WB_/Saarikangas 2021-07-15 20h58m50s_Chemiluminescence.jpg]

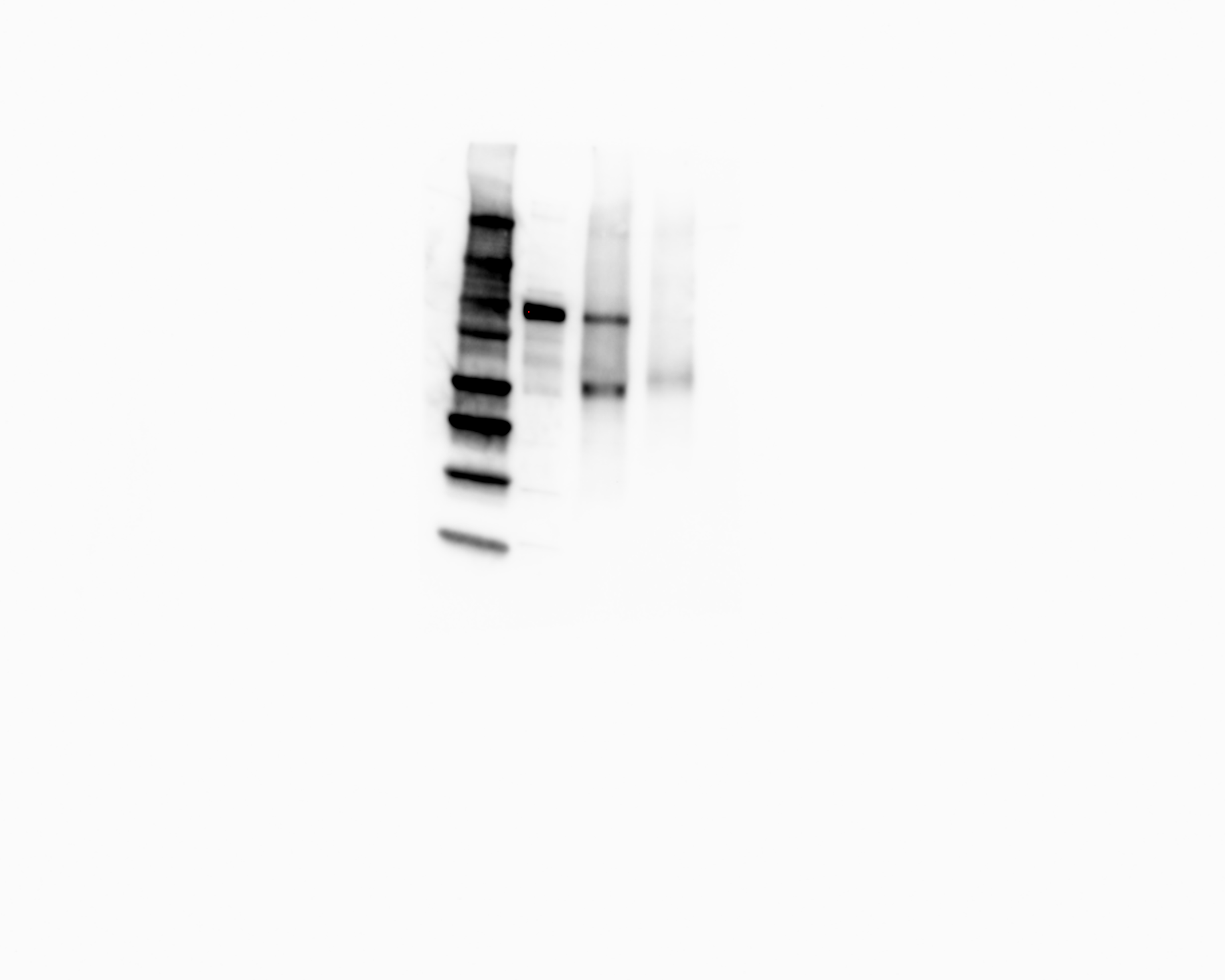

Supplement: Figure 4—figure supplement 1—source data 1. [file elife-92748-fig4-figsupp1-data1.zip › Figure 4 D-figure Supplemental-Source data-anti-Abba WB_/Saarikangas 2021-07-15 20h58m50s_Chemiluminescence.tif]
