# Supplementary material for: Identification of novel human microcephaly-linked protein Mtss2 that mediates cortical progenitor cell division and corticogenesis through Nedd9-RhoA
Source: eLife. 2025 Jul 23;13:RP92748. doi: 10.7554/eLife.92748 (PMC12286603; doi:10.7554/eLife.92748)
Supplement: Supplementary file 1. [file elife-92748-supp1.pdf]

HYBRIGENICS

services

Results Summary

Mouse ABBA-1
# vs Mouse Embryo Brain RP2

Screen Parameters

Nature	cDNA
Reference Bait Fragment	Mouse ABBA-1 (1-715) ; hgx2114v1
Prey Library	Mouse Embryo Brain RP2
Vector(s)	pB27 (N-LexA-bait-C fusion) + pB66 (N-GAL4-bait-C fusion)
Processed Clones	15 (pB27) + 269 (pB66) + 87 (pB66)
Analyzed Interactions	91 millions (pB27) + 23.2 millions (pB66) + 8.12 millions (pB66)
3AT Concentration	0.0 mM (pB27) + 0.0 mM (pB66) + 0.0 mM (pB66)

Global PBS®

Global PBS (for Interactions represented in the Screen)		Nb	%
A	Very high confidence in the interaction	0	0.0%
B	High confidence in the interaction	1	1.0%
C	Good confidence in the interaction	3	2.9%
D	Moderate confidence in the interaction<br>This category is the most difficult to interpret because it mixes two classes of interactions :<br>- False-positive interactions<br>- Interactions hardly detectable by the Y2H technique (low representation of the mRNA in the library, prey folding, prey toxicity in yeast)	95	93.1%
E	Interactions involving highly connected prey domains, warning of non-specific interaction. The threshold for high connectivity is 10 for screens with Human, Mouse, Drosophila and Arabidopsis and 6 for all other organisms. They can be classified in different categories:<br>- Prey proteins that are known to be highly connected due to their biological function<br>- Proteins with a prey interacting domain that contains a known protein interaction motif or a biochemically promiscuous motif	1	1.0%
F	Experimentally proven technical artifacts	2	2.0%
Non Appliable			
N/A	The PBS is a score that is automatically computed through algorithms and cannot be attributed for the following reasons :<br>- All the fragments of the same reference CDS are antisens<br>- The 5p sequence is missing<br>- All the fragments of the same reference CDS are either all OOF1 or all OOF2<br>- All the fragments of the same reference CDS lie in the 5' or 3' UTR		

Prey Fragment Analysis

Symbols	Means
✳	The fragment contains the full length CDS
↶	Fragment is fully in 5' UTR
↷	Fragment is fully in 3' UTR
✗	Fragment contains at least one In Frame STOP codon
[NR]	Fragment was found to be non relevant (poor quality, high N density)
IF<br>OOF1<br>OOF2	With regard to the theoretical frame of each corresponding CDS (GeneBank),fragments are cloned in frame (IF) if they are in the same frame as Gal4AD.In general, polypeptides synthesized from OOF fragments are not considered of biological interest, unless found together with another frame. However, some of the proteins expressed from an OOF fragment can be translated in the correct frame,due to the existence of natural frame-shift events during translation in yeast
??	Unidentified frame when :<br>- The clone sequence is antisense<br>- The 5p sequence is missing
N	Antisens
Start...Stop	Position of the 5p and 3p prey fragment ends, relative to the position of the ATG start codon (A=0)

Clone Name	Type Seq	Gene Name (Best Match)	Start..Stop (nt)	Frame	Sens	%Id 5p	%Id 3p	PBS
pB66_A-88	5p/3p	Mus musculus - ABBA-1	-52..780	IF		99.0	99.8	D
pB66_A-185	5p/3p	Mus musculus - AU016693	3053..3797 ↷ ✗	OOF2		99.8	99.8	N/A
pB66_A-261	3p	Mus musculus - Atf2	..2101	??	N		ALU 98.1	N/A
pB66_B-93	5p/3p	Mus musculus - BC018245	2873..1560	??	N	69.1	99.1	N/A
pB66_A-76	5p/3p	Mus musculus - BC018245	2873..1560	??	N	78.4	100.0	N/A
pB66_A-165	5p/3p	Mus musculus - BC066049	2560..2089	??	N	100.0	100.0	N/A
pB66_A-120	5p/3p	Mus musculus - BC066049	2569..2258	??	N	99.7	99.7	N/A
pB66_A-18	5p/3p	Mus musculus - BC066049	2569..2258	??	N	99.7	99.7	N/A
pB66_A-169	5p/3p	Mus musculus - BC086774	1438..2127 ✗	OOF1		85.2	99.3	N/A
pB66_A-254	3p	Mus musculus - Cdca5	..49	??	N		78.5	N/A
pB66_B-80	5p/3p	Mus musculus - Cdca5	465..-48	??	N	92.7	90.9	N/A
pB66_A-212	5p/3p	Mus musculus - Cdca5	465..-101	??	N	93.7	99.6	N/A
pB66_A-202	5p/3p	Mus musculus - Cdca5	550..30	??	N	73.6	100.0	N/A
pB66_B-24	5p/3p	Mus musculus - Cdca5	551..49	??	N	92.0	100.0	N/A
pB66_A-209	5p/3p	Mus musculus - Cdca5	552..30	??	N	91.4	99.8	N/A
pB66_A-174	5p/3p	Mus musculus - Cdca5	552..30	??	N	100.0	100.0	N/A
pB66_A-173	5p/3p	Mus musculus - Cdca5	552..30	??	N	100.0	100.0	N/A
pB66_A-176	5p/3p	Mus musculus - Cdca5	552..30	??	N	100.0	100.0	N/A
pB66_B-53	5p/3p	Mus musculus - Cdca5	552..30	??	N	99.2	100.0	N/A
pB66_A-143	5p/3p	Mus musculus - Cdca5	552..30	??	N	100.0	100.0	N/A
pB66_A-205	5p/3p	Mus musculus - Cdca5	552..30	??	N	100.0	100.0	N/A
pB66_B-89	5p/3p	Mus musculus - Cdca5	552..30	??	N	100.0	100.0	N/A
pB66_A-225	5p/3p	Mus musculus - Cdca5	552..30	??	N	100.0	100.0	N/A
pB66_A-177	5p/3p	Mus musculus - Cdca5	552..30	??	N	100.0	100.0	N/A
pB66_A-231	5p/3p	Mus musculus - Cdca5	552..49	??	N	99.6	100.0	N/A
pB66_B-30	5p/3p	Mus musculus - Cdca5	552..49	??	N	99.6	99.8	N/A
pB66_B-41	5p/3p	Mus musculus - Cdca5	552..49	??	N	99.2	100.0	N/A
pB66_A-223	5p/3p	Mus musculus - Cdca5	552..49	??	N	98.1	100.0	N/A
pB66_A-115	5p/3p	Mus musculus - Cdca5	552..49	??	N	100.0	100.0	N/A

Clone Name	Type Seq	Gene Name (Best Match)	Start..Stop (nt)	Frame	Sens	%Id 5p	%Id 3p	PBS
pB66_A-70	5p/3p	Mus musculus - Cdca5	552..49	??	N	100.0	100.0	N/A
pB66_A-69	5p/3p	Mus musculus - Cdca5	552..49	??	N	100.0	100.0	N/A
pB66_A-51	5p/3p	Mus musculus - Cdca5	552..49	??	N	100.0	100.0	N/A
pB66_A-30	5p/3p	Mus musculus - Cdca5	552..49	??	N	100.0	100.0	N/A
pB66_A-196	5p/3p	Mus musculus - Cdca5	552..49	??	N	93.2	99.6	N/A
pB66_A-127	5p/3p	Mus musculus - Cdca5	552..30	??	N	100.0	100.0	N/A
pB66_A-162	5p/3p	Mus musculus - Cdca5	555..-33	??	N	100.0	100.0	N/A
pB66_A-179	5p/3p	Mus musculus - Cdca5	558..74	??	N	100.0	100.0	N/A
pB66_A-106	5p/3p	Mus musculus - Cdca5	558..-23	??	N	100.0	100.0	N/A
pB66_A-262	5p/3p	Mus musculus - Cdca5	558..-23	??	N	99.1	100.0	N/A
pB66_A-35	5p/3p	Mus musculus - Cdca5	561..-29	??	N	100.0	100.0	N/A
pB66_A-259	5p/3p	Mus musculus - Cdca5	561..-29	??	N	89.5	99.7	N/A
pB66_A-112	5p/3p	Mus musculus - Cdca5	561..-29	??	N	100.0	100.0	N/A
pB66_A-249	5p/3p	Mus musculus - Cdca5	657..325	??	N	99.4	100.0	N/A
pB66_A-110	5p/3p	Mus musculus - Cdca5	657..325	??	N	100.0	100.0	N/A
pB66_B-26	3p	Mus musculus - Cdca5	1057..554	??	N		100.0	N/A
pB66_A-215	3p	Mus musculus - Cdca5	1057..554	??	N		100.0	N/A
pB66_A-217	3p	Mus musculus - Cdca5	1057..554	??	N		100.0	N/A
pB66_A-226	3p	Mus musculus - Cdca5	1068..554	??	N		100.0	N/A
pB66_A-198	3p	Mus musculus - Cdca5	1076..554	??	N		100.0	N/A
pB66_A-272	3p	Mus musculus - Cdca5	1078..554	??	N		99.8	N/A
pB66_A-283	3p	Mus musculus - Cdca5	1140..560	??	N		99.5	N/A
pB66_A-166	3p	Mus musculus - Cep70	..1335	??	N		98.8	N/A
pB66_B-91	5p/3p	Mus musculus - Cep70	2245..1335	??	N	99.0	98.6	N/A
pB66_B-33	5p/3p	Mus musculus - Cep70	2245..1335	??	N	98.1	98.8	N/A
pB66_B-76	5p/3p	Mus musculus - Cep70	2245..1335	??	N	99.0	98.6	N/A
pB66_A-83	5p/3p	Mus musculus - Cep70	2245..1335	??	N	99.1	99.5	N/A
pB66_A-94	5p/3p	Mus musculus - Cep70	2245..1335	??	N	99.2	99.3	N/A
pB66_A-164	5p/3p	Mus musculus - Cep70	2245..1335	??	N	99.2	99.0	N/A
pB66_A-218	5p/3p	Mus musculus - Cep70	2245..1335	??	N	97.9	98.5	N/A
pB66_A-27	5p/3p	Mus musculus - Cep70	2245..1335	??	N	99.4	99.0	N/A
pB66_A-159	5p/3p	Mus musculus - Cep70	2245..1335	??	N	98.8	93.2	N/A
pB66_B-72	5p/3p	Mus musculus - Cep70	2245..1335	??	N	98.4	97.5	N/A
pB66_B-38	5p/3p	Mus musculus - Cep70	2218..1160	??	N	97.4	81.6	N/A
pB66_B-16	5p/3p	Mus musculus - Cops5	78..827	IF		74.5	100.0	F
pB66_B-55	5p/3p	Mus musculus - Csnk1g3	1598..2110	OOF2		100.0	100.0	N/A
pB66_A-211	5p/3p	Mus musculus - D15Wsu75e	410..1300	OOF2		97.9	99.5	N/A
pB66_B-46	5p/3p	Mus musculus - D15Wsu75e	452..1000	OOF2		100.0	100.0	N/A
pB66_B-52	5p/3p	Mus musculus - D15Wsu75e	473..1040	OOF2		99.8	100.0	N/A
pB66_B-54	5p/3p	Mus musculus - D15Wsu75e	473..853	OOF2		99.0	100.0	N/A
pB66_A-154	5p/3p	Mus musculus - D15Wsu75e	473..853	OOF2		100.0	100.0	N/A
pB66_B-77	5p/3p	Mus musculus - D15Wsu75e	473..853	OOF2		89.8	100.0	N/A
pB66_B-44	5p/3p	Mus musculus - D15Wsu75e	473..853	OOF2		96.1	100.0	N/A
pB66_B-90	5p/3p	Mus musculus - D15Wsu75e	479..847	OOF2		98.6	100.0	N/A
pB66_B-82	5p/3p	Mus musculus - D15Wsu75e	479..847	OOF2		96.5	100.0	N/A
pB66_A-22	5p/3p	Mus musculus - D15Wsu75e	479..847	OOF2		100.0	100.0	N/A
pB66_A-40	5p/3p	Mus musculus - D15Wsu75e	479..847	OOF2		98.9	98.1	N/A
pB66_A-11	5p/3p	Mus musculus - D15Wsu75e	479..847	OOF2		100.0	100.0	N/A
pB66_A-265	5p/3p	Mus musculus - Dach2	2398..1352	??	N	86.2	97.1	N/A
pB66_B-71	5p/3p	Mus musculus - Dach2	2399..1352	??	N	98.5	95.5	N/A
pB66_B-19	5p/3p	Mus musculus - Dach2	2399..1352	??	N	99.0	96.1	N/A

Clone Name	Type Seq	Gene Name (Best Match)	Start..Stop (nt)	Frame	Sens	%Id 5p	%Id 3p	PBS
pB66 B-84	5p	Mus musculus - Dach2	2399	??	N	96.4		N/A
pB66 A-178	5p/3p	Mus musculus - Dach2	2399..1352	??	N	99.2	95.8	N/A
pB66 A-234	5p/3p	Mus musculus - Dach2	2399..1352	??	N	98.8	95.6	N/A
pB66 A-28	5p/3p	Mus musculus - Dach2	2399..1352	??	N	98.7	96.6	N/A
pB66 A-17	5p/3p	Mus musculus - Dach2	2399..1352	??	N	98.7	97.1	N/A
pB66 A-21	5p/3p	Mus musculus - Dgcr8	1254..716	??	N	100.0	99.8	N/A
pB66 B-17	5p/3p	Mus musculus - Dip2b	1796..868	??	N	81.4	83.3	B
pB66 A-253	3p	Mus musculus - Fbxw11	..1828	??			99.7	B
pB27 A-14	5p/3p	Mus musculus - Fbxw11	699..1844	IF		99.4	100.0	B
pB27 A-10	5p/3p	Mus musculus - Fbxw11	699..1844	IF		99.7	100.0	B
pB27 A-9	5p/3p	Mus musculus - Fbxw11	699..1844	IF		99.3	100.0	B
pB27 A-17	5p/3p	Mus musculus - Fbxw11	699..1844	IF		99.6	99.3	B
pB27 A-4	5p/3p	Mus musculus - Fbxw11	699..1828	IF		99.4	99.7	B
pB27 A-16	5p/3p	Mus musculus - Fbxw11	711..1810	IF		100.0	100.0	B
pB27 A-11	5p/3p	Mus musculus - Fbxw11	711..1810	IF		98.8	99.8	B
pB27 A-18	5p/3p	Mus musculus - Fbxw11	711..1810	IF		99.6	99.8	B
pB66 A-229	5p/3p	Mus musculus - Fbxw11	711..1810	IF		95.5	100.0	B
pB27 A-7	5p/3p	Mus musculus - Fbxw11	711..1810	IF		99.7	100.0	B
pB27 A-12	5p/3p	Mus musculus - Glrx3	363..960	IF		89.3	89.9	D
pB66 B-25	5p/3p	Mus musculus - HTC FLI	16..398	OOF1		89.3	100.0	N/A
pB66 A-213	5p/3p	Mus musculus - HTC FLI	16..398	OOF1		99.2	100.0	N/A
pB66 B-83	5p/3p	Mus musculus - HTC FLI	16..398	OOF1		100.0	100.0	N/A
pB66 A-183	5p/3p	Mus musculus - HTC FLI	16..398	OOF1		100.0	100.0	N/A
pB66 A-180	5p/3p	Mus musculus - HTC FLI	16..398	OOF1		100.0	100.0	N/A
pB66 A-102	5p/3p	Mus musculus - HTC FLI	16..398	OOF1		100.0	100.0	N/A
pB66 A-57	5p/3p	Mus musculus - HTC FLI	16..398	OOF1		100.0	100.0	N/A
pB66 B-45	5p/3p	Mus musculus - Isca1	1141..1836	OOF1		99.6	99.8	N/A
pB66 B-3	5p/3p	Mus musculus - Isca1	1141..1836	OOF1		99.9	100.0	N/A
pB66 A-117	5p/3p	Mus musculus - Isca1	1141..1682	OOF1		100.0	100.0	N/A
pB66 A-9	5p/3p	Mus musculus - Isca1	1141..1682	OOF1		100.0	100.0	N/A
pB66 A-237	5p/3p	Mus musculus - Isca1	1141..1682	OOF1		99.8	97.6	N/A
pB66 A-150	5p/3p	Mus musculus - Isca1	1141..1682	OOF1		99.3	100.0	N/A
pB66 A-258	5p/3p	Mus musculus - Isca1	1141..1682	OOF1		99.8	93.7	N/A
pB66 A-46	5p/3p	Mus musculus - Isca1	1141..1836	OOF1		99.7	99.8	N/A
pB66 A-84	5p/3p	Mus musculus - Isca1	1141..1836	OOF1		99.8	100.0	N/A
pB66 A-232	5p	Mus musculus - Isca1	1141	OOF1		97.1		N/A
pB66 A-247	5p/3p	Mus musculus - Isca1	1141..1682	OOF1		82.5	99.8	N/A
pB66 A-5	5p/3p	Mus musculus - Isca1	1141..1836	OOF1		97.3	96.9	N/A
pB66 A-238	5p/3p	Mus musculus - Isca1	1142..1682	OOF2		78.0	100.0	N/A
pB66_A-149	5p/3p	Mus musculus - Isca1	1198..1470	OOF1		98.2	100.0	N/A
pB66 A-53	5p/3p	Mus musculus - Isca1	1198..1470	OOF1		99.3	100.0	N/A
pB66 B-8	5p/3p	Mus musculus - Isca1	1198..1470	OOF1		99.3	96.0	N/A
pB66 A-107	5p/3p	Mus musculus - Isca1	1198..1470	OOF1		100.0	100.0	N/A
pB66 A-60	5p/3p	Mus musculus - Isca1	1198..1470	OOF1		100.0	100.0	N/A
pB66 A-191	5p/3p	Mus musculus - Isca1	1198..1470	OOF1		100.0	99.6	N/A
pB66 A-90	5p/3p	Mus musculus - Kalrn	105..730	IF		99.8	99.5	D
pB66 B-40	5p/3p	Mus musculus - LOC100042092	1676..1338	??	N	92.0	97.6	N/A
pB66 A-85	5p/3p	Mus musculus - LOC100042229	-1922..-1248	OOF2		79.5	92.5	N/A

Clone Name	Type Seq	Gene Name (Best Match)	Start..Stop (nt)	Frame	Sens	%Id 5p	%Id 3p	PBS
pB66_A-65	5p/3p	Mus musculus - LOC100045522	4321..4843	OOF1		99.8	85.7	N/A
pB66_A-138	5p/3p	Mus musculus - LOC668039	4733..3456	??	N	98.8	99.7	N/A
pB66_A-228	5p/3p	Mus musculus - Lrrn1	1922..1188	??	N	93.6	99.7	N/A
pB66_A-152	5p/3p	Mus musculus - Lrrn1	1922..1188	??	N	100.0	99.7	N/A
pB66_A-116	5p/3p	Mus musculus - Lrrn1	1922..1188	??	N	100.0	100.0	N/A
pB66_A-195	5p/3p	Mus musculus - Mll1	16440..15614	??	N	95.0	98.0	N/A
pB66_A-182	5p/3p	Mus musculus - Mtss1	-67..777	IF		98.0	97.9	D
pB66_B-69	5p/3p	Mus musculus - Napg	1228..1803	OOF1		99.7	100.0	N/A
pB66_A-142	5p/3p	Mus musculus - Napg	1228..1803	OOF1		99.0	100.0	N/A
pB66_A-157	5p/3p	Mus musculus - Nedd9	2415..3591	IF		99.2	99.5	C
pB66_B-22	5p/3p	Mus musculus - Nedd9	2463..2937	IF		96.8	100.0	C
pB66_A-252	5p/3p	Mus musculus - Nedd9	2463..2937	IF		100.0	100.0	C
pB66_A-263	5p/3p	Mus musculus - Nedd9	2463..2937	IF		92.7	95.7	C
pB66_B-62	5p/3p	Mus musculus - Ngfr	1911..1336	??	N	83.5	99.0	N/A
pB66_A-93	5p/3p	Mus musculus - Nrxn3	3796..4338	OOF1		99.1	100.0	N/A
pB66_A-248	5p/3p	Mus musculus - Nrxn3	3796..4338	OOF1		91.4	97.8	N/A
pB66_A-13	5p/3p	Mus musculus - Nrxn3	3796..4338	OOF1		98.7	100.0	N/A
pB66_B-94	5p/3p	Mus musculus - Nrxn3	3796..4338	OOF1		95.9	99.8	N/A
pB66_B-64	5p/3p	Mus musculus - Nrxn3	3796..4338	OOF1		96.3	98.3	N/A
pB66_B-34	5p/3p	Mus musculus - Nrxn3	3796..4338	OOF1		98.2	100.0	N/A
pB66_A-50	5p/3p	Mus musculus - Nrxn3	3796..4338	OOF1		95.9	100.0	N/A
pB66_A-128	5p/3p	Mus musculus - Nrxn3	3796..4338	OOF1		98.5	100.0	N/A
pB66_A-34	5p/3p	Mus musculus - Nrxn3	3796..4338	OOF1		99.3	100.0	N/A
pB66_B-11	5p/3p	Mus musculus - Nrxn3	3796..4338	OOF1		99.4	99.8	N/A
pB66_A-222	5p/3p	Mus musculus - Nsd1	4839..5813	IF		98.5	99.8	D
pB27_A-6	5p/3p	Mus musculus - Nsd1	4839..5813	IF		99.9	100.0	D
pB66_A-194	5p/3p	Mus musculus - Otx2	51..283	IF		90.6	99.1	C
pB66_A-278	5p/3p	Mus musculus - Otx2	51..283	IF		95.7	97.9	C
pB66_A-250	5p/3p	Mus musculus - Otx2	51..283	IF		97.0	99.6	C
pB66_A-168	5p/3p	Mus musculus - Otx2	54..283	IF		92.5	99.6	C
pB66_A-276	5p/3p	Mus musculus - Pax3	397..927	OOF1		82.5	93.2	N/A
pB66_A-245	3p	Mus musculus - Pax6	..1054	??			99.5	F
pB66_B-61	3p	Mus musculus - Pax6	..933	??			99.8	F
pB66_B-51	3p	Mus musculus - Pax6	..933	??			99.2	F
pB66_B-47	3p	Mus musculus - Pax6	..858	??			99.7	F
pB66_A-269	3p	Mus musculus - Pax6	..858	??			99.1	F
pB66_A-47	5p/3p	Mus musculus - Pax6	30..890	IF		93.7	99.1	F
pB66_B-35	5p/3p	Mus musculus - Pax6	42..858	IF		93.7	98.4	F
pB66_A-134	5p/3p	Mus musculus - Pax6	105..881	IF		94.0	100.0	F
pB66_A-155	5p/3p	Mus musculus - Pax6	177..1005	IF		99.1	97.8	F
pB66_A-75	5p/3p	Mus musculus - Pax6	225..933	IF		99.6	99.5	F
pB66_A-216	3p	Mus musculus - Phldb2	..3592	??	N		98.8	N/A
pB66_A-91	5p/3p	Mus musculus - Phldb2	4723..3592	??	N	97.9	99.3	N/A
pB66_A-268	5p/3p	Mus musculus - Phldb2	4723..3592	??	N	95.1	98.9	N/A
pB66_A-24	5p/3p	Mus musculus - Ptprs	5870..6218	OOF2		99.1	99.7	N/A
pB66_A-273	3p	Mus musculus - Ranbp9	..1351	??			99.8	N/A
pB66_B-10	5p/3p	Mus musculus - Rfk	1494..788	??	N	86.2	ALU 94.1	N/A
pB66_A-54	5p/3p	Mus musculus - Rsad1	2650..3439	OOF1		99.4	99.4	N/A

Clone Name	Type Seq	Gene Name (Best Match)	Start..Stop (nt)	Frame	Sens	%Id 5p	%Id 3p	PBS
pB66_B-79	5p/3p	Mus musculus - Sephs2	1764..2070 ✗	IF		ALU 95.7	64.1	N/A
pB66_B-39	5p/3p	Mus musculus - Sephs2	1764..2070 ✗	IF		ALU 95.4	ALU 65.3	N/A
pB66_A-78	5p/3p	Mus musculus - Sephs2	1764..2069 ✗	IF		ALU 99.3	ALU 64.1	N/A
pB66_A-108	5p/3p	Mus musculus - Sqol2	24..552	IF		100.0	100.0	D
pB66_B-28	5p/3p	Mus musculus - Slc2a3	2167..2754 ✗	OOF1		91.2	99.8	N/A
pB66_B-88	5p/3p	Mus musculus - Tbl1xr1	3025..3490 ✗	OOF1		92.5	99.6	N/A
pB66_A-203	5p/3p	Mus musculus - Tbl1xr1	3025..3490 ✗	OOF1		92.3	100.0	N/A
pB66_A-87	5p/3p	Mus musculus - Tbl1xr1	3025..3490 ✗	OOF1		99.6	100.0	N/A
pB66_A-114	5p/3p	Mus musculus - Tbl1xr1	3025..3490 ✗	OOF1		95.0	100.0	N/A
pB66_A-32	5p/3p	Mus musculus - Tbl1xr1	3025..3490 ✗	OOF1		100.0	100.0	N/A
pB27_A-13	5p/3p	Mus musculus - Trim28	1021..509	??	N	100.0	100.0	N/A
pB66_B-75	5p	Mus musculus - Uap1	390..-18	??	N	93.2		N/A
pB66_B-73	3p	Mus musculus - Uap1	806..393	??	N		99.5	N/A
pB66_A-210	5p/3p	Mus musculus - Whsc1	424..-118	??	N	97.8	97.6	N/A
pB66_A-190	5p/3p	Mus musculus - Whsc1	424..-118	??	N	97.1	97.6	N/A
pB66_B-29	5p/3p	Mus musculus - Whsc1	424..134	??	N	91.3	98.6	N/A
pB66_A-146	5p/3p	Mus musculus - Whsc1	424..134	??	N	100.0	100.0	N/A
pB66_A-96	5p/3p	Mus musculus - Whsc1	424..-118	??	N	97.8	97.6	N/A
pB66_A-92	5p/3p	Mus musculus - Whsc1	424..134	??	N	100.0	100.0	N/A
pB66_A-97	5p/3p	Mus musculus - Whsc1	424..-227	??	N	87.5	86.2	N/A
pB66_A-284	5p/3p	Mus musculus - Wwtr1	1913..2759 ✗	OOF2		92.4	98.9	N/A
pB66_A-188	5p/3p	Mus musculus - Wwtr1	1913..2759 ✗	OOF2		94.7	98.8	N/A
pB66_A-181	5p/3p	Mus musculus - Wwtr1	1913..2759 ✗	OOF2		88.4	99.5	N/A
pB27_A-5	5p/3p	Mus musculus - Zbtb16	1326..2153 ✗	IF		98.5	99.3	E
pB27_A-8	5p/3p	Mus musculus - Zbtb16	1326..2153 ✗	IF		99.4	97.9	E
pB27_A-15	5p/3p	Mus musculus - Zbtb16	1326..2153 ✗	IF		95.9	71.2	E
pB66_A-61	5p/3p	Mus musculus - Zmym4	1576	OOF1		65.5	99.5	N/A
pB66_A-282	5p/3p	Mus musculus - Zmym4	1576	OOF1		66.7	93.2	N/A
pB66_A-38	5p/3p	Mus musculus - Zmym4	4456..3078	??	N	94.7	99.8	N/A
pB66_A-126	5p/3p	Mus musculus - Znf512b	5540..5124	??	N	100.0	100.0	N/A
pB66_A-255	3p	Mus musculus - No Match (no match found in GenBank)	..560 [NR]	??			100.0	N/A
pB66_B-36	5p/3p	Mus musculus - No Match (no match found in GenBank)		??		100.0	100.0	N/A
pB66_A-266	5p/3p	Mus musculus - GenMatch GID: 47825183	-1	IF		80.5	94.5	D
pB66_B-95	5p/3p	Mus musculus - GenMatch GID: 47825183	-1	IF		91.0	93.8	D
pB66_B-58	5p/3p	Mus musculus - GenMatch GID: 47825183	-1	IF		68.1	94.1	D
pB66_A-71	3p	Mus musculus - GenMatch GID: 56236271	..568	??			100.0	N/A
pB66_A-241	5p/3p	Mus musculus - GenMatch GID: 34787446	-1..497 ✗	IF		100.0	82.2	D
pB66_A-251	5p/3p	Mus musculus - GenMatch GID: 26006611	-1..478 ✗	IF		100.0	75.4	D
pB66_A-279	5p/3p	Mus musculus - GenMatch GID: 77404072	-1..500 ✗	IF		100.0	89.8	D
pB66_A-281	5p/3p	Mus musculus - GenMatch GID: 24459826	-1..393 ✗	IF		100.0	89.3	D
pB66_B-12	5p/3p	Mus musculus - GenMatch GID: 77404072	-1..502 ✗	IF		99.4	100.0	D

Clone Name	Type Seq	Gene Name (Best Match)	Start..Stop (nt)	Frame	Sens	%Id 5p	%Id 3p	PBS
pB66_A-161	5p/3p	Mus musculus - GenMatch GID: 77404072	-1..502	IF		99.8	99.8	
pB66_B-4	5p/3p	Mus musculus - GenMatch GID: 33285224	-1..1006	IF		100.0	92.5	
pB66_A-89	5p/3p	Mus musculus - GenMatch GID: 20068727	-1	IF		100.0	82.6	
pB66_A-26	5p/3p	Mus musculus - GenMatch GID: 20068727	-1..776	IF		100.0	57.3	
pB66_B-6	5p/3p	Mus musculus - GenMatch GID: 38093845	-1..687	IF		100.0	85.3	
pB66_B-20	5p/3p	Mus musculus - GenMatch GID: 81295619	-1	IF		ALU 92.7	93.8	
pB66_A-8	5p/3p	Mus musculus - GenMatch GID: 25955801	-1..579	IF		99.8	99.8	
pB66_A-56	5p/3p	Mus musculus - GenMatch GID: 39652666	-1..576	IF		100.0	99.8	
pB66_A-29	5p/3p	Mus musculus - GenMatch GID: 39652666	-1..576	IF		99.8	100.0	
pB66_A-73	5p/3p	Mus musculus - GenMatch GID: 53378873	-1..582	IF		100.0	58.3	
pB66_A-239	5p/3p	Mus musculus - GenMatch GID: 21671875	-1..745	IF		100.0	88.9	
pB66_A-257	5p/3p	Mus musculus - GenMatch GID: 84794837	-1..298	IF		100.0	89.5	
pB66_A-55	5p/3p	Mus musculus - GenMatch GID: 37718670	-1..399	IF		95.2	100.0	
pB66_A-103	5p/3p	Mus musculus - GenMatch GID: 27356737	-1..549	IF		99.8	100.0	
pB66_B-70	5p/3p	Mus musculus - GenMatch GID: 51339134	-1..410	IF		99.0	100.0	
pB66_A-172	5p/3p	Mus musculus - GenMatch GID: 51339134	-1..410	IF		100.0	100.0	
pB66_A-104	5p/3p	Mus musculus - GenMatch GID: 58652300	-1..644	IF		99.1	100.0	
pB66_A-1	5p/3p	Mus musculus - GenMatch GID: 76683163	-1..323	IF		100.0	83.1	
pB66_B-23	5p/3p	Mus musculus - GenMatch GID: 26006611	-1..490	IF		100.0	84.7	
pB66_B-86	5p/3p	Mus musculus - GenMatch GID: 47078043	-1..456	IF		100.0	87.5	
pB66_A-207	5p/3p	Mus musculus - GenMatch GID: 47078043	-1..436	IF		100.0	82.5	
pB66_A-214	5p/3p	Mus musculus - GenMatch GID: 58652300	-1	IF		100.0	66.7	
pB66_A-7	5p/3p	Mus musculus - GenMatch GID: 81295619	-1	IF		ALU 92.8	93.8	
pB66_B-87	5p/3p	Mus musculus - GenMatch GID: 58332941	-1..1186	IF		100.0	85.5	
pB66_A-135	5p/3p	Mus musculus - GenMatch GID: 33438711	-1..737	IF		99.7	99.4	
pB66_A-208	5p/3p	Mus musculus - GenMatch GID: 33438711	-1..737	IF		99.0	99.1	
pB66_A-256	5p/3p	Mus musculus - GenMatch GID: 33438711	-1..737	IF		99.7	98.8	
pB66_A-260	5p/3p	Mus musculus - GenMatch GID: 33438711	-1..737	IF		99.7	99.2	
pB66_A-119	5p/3p	Mus musculus - GenMatch GID: 33438711	-1..737	IF		100.0	99.7	
pB66_A-45	5p/3p	Mus musculus - GenMatch GID: 33438711	-1..737	IF		99.9	99.2	

Clone Name	Type Seq	Gene Name (Best Match)	Start..Stop (nt)	Frame	Sens	%Id 5p	%Id 3p	PBS
pB66_A-86	5p/3p	Mus musculus - GenMatch GID: 81295619	-1	IF		ALU 92.8	93.8	D
pB66_A-98	3p	Mus musculus - GenMatch GID: 50839068	..373	??			100.0	N/A
pB66_A-101	5p/3p	Mus musculus - GenMatch GID: 81295619	-1	IF		ALU 92.5	54.4	D
pB66_B-66	5p/3p	Mus musculus - GenMatch GID: 24459826	-1..393	IF		100.0	87.3	D
pB66_A-59	5p/3p	Mus musculus - GenMatch GID: 61741006	-1..822	IF		100.0	100.0	D
pB66_A-158	5p/3p	Mus musculus - GenMatch GID: 34013588	-1..1230	IF		100.0	100.0	D
pB66_A-64	5p/3p	Mus musculus - GenMatch GID: 34013588	-1..1230	IF		99.9	85.2	D
pB66_A-118	5p/3p	Mus musculus - GenMatch GID: 28268663	-1..790	IF		100.0	100.0	D
pB66_A-105	5p/3p	Mus musculus - GenMatch GID: 72255689	-1..534	IF		100.0	100.0	D
pB66_B-74	5p/3p	Mus musculus - GenMatch GID: 39725818	-1..416	IF		100.0	92.1	D
pB66_A-10	5p/3p	Mus musculus - GenMatch GID: 20068621	-1..880	IF		100.0	91.8	D
pB66_B-18	5p/3p	Mus musculus - GenMatch GID: 45825198	-1	IF		94.5	ALU 64.0	D
pB66_B-7	5p/3p	Mus musculus - GenMatch GID: 45825198	-1	IF		85.9	ALU 64.0	D
pB66_A-49	5p/3p	Mus musculus - GenMatch GID: 45825198	-1	IF		91.8	ALU 61.3	D
pB66_A-131	5p/3p	Mus musculus - GenMatch GID: 45825198	-1	IF		98.0	ALU 62.7	D
pB66_B-5	5p/3p	Mus musculus - GenMatch GID: 23379824	-1..537	IF		100.0	100.0	D
pB66_A-201	5p/3p	Mus musculus - GenMatch GID: 23379824	-1..537	IF		99.4	100.0	D
pB66_A-184	5p/3p	Mus musculus - GenMatch GID: 23379824	-1..537	IF		100.0	99.8	D
pB66_A-12	5p/3p	Mus musculus - GenMatch GID: 47078043	-1..462	IF		100.0	84.1	D
pB66_A-15	5p/3p	Mus musculus - GenMatch GID: 81295619	-1	IF		ALU 92.9	93.8	D
pB66_A-137	5p/3p	Mus musculus - GenMatch GID: 73120244	-1..500	IF		100.0	100.0	D
pB66_A-16	5p/3p	Mus musculus - GenMatch GID: 81295619	-1	IF		ALU 93.1	93.8	D
pB66_A-235	5p/3p	Mus musculus - GenMatch GID: 20068621	-1..873	IF		96.3	99.1	D
pB66_A-267	3p	Mus musculus - GenMatch GID: 20068621	6..305	IF			99.3	D
pB66_A-140	5p/3p	Mus musculus - GenMatch GID: 85060584	-1..275	IF		99.7	65.0	D
pB66_A-139	5p/3p	Mus musculus - GenMatch GID: 85060584	-1..275	IF		99.1	65.0	D
pB66_A-25	5p/3p	Mus musculus - GenMatch GID: 81778246	-1..517	IF		100.0	92.6	D
pB66_A-236	5p/3p	Mus musculus - GenMatch GID: 38564394	-1..438	IF		99.8	98.2	D
pB66_A-243	5p/3p	Mus musculus - GenMatch GID: 22204310	-1..850	IF		98.7	99.9	D
pB66_A-171	5p/3p	Mus musculus - GenMatch GID: 81295619	-1	IF		ALU 92.6	93.8	D

Clone Name	Type Seq	Gene Name (Best Match)	Start..Stop (nt)	Frame	Sens	%Id 5p	%Id 3p	PBS
pB66_A-122	5p/3p	Mus musculus - GenMatch GID: 76362883	-1..574	IF		99.8	99.8	D
pB66_B-78	5p/3p	Mus musculus - GenMatch GID: 76362883	-1..574	IF		100.0	99.7	D
pB66_A-125	5p/3p	Mus musculus - GenMatch GID: 76362883	-1..574	IF		99.1	99.8	D
pB66_A-280	3p	Mus musculus - GenMatch GID: 76362883	-1..573	IF			99.5	D
pB66_A-285	5p/3p	Mus musculus - GenMatch GID: 76362883	-1..574	IF		97.6	99.1	D
pB66_A-31	5p/3p	Mus musculus - GenMatch GID: 76362883	-1..574	IF		98.8	99.8	D
pB66_A-160	5p/3p	Mus musculus - GenMatch GID: 76362883	-1..574	IF		99.7	99.5	D
pB66_A-33	5p/3p	Mus musculus - GenMatch GID: 76362883	-1..574	IF		100.0	100.0	D
pB66_A-187	5p/3p	Mus musculus - GenMatch GID: 20068621	-1..874	IF		100.0	71.7	D
pB66_A-151	5p/3p	Mus musculus - GenMatch GID: 81295619	-1	IF		ALU 92.5	93.8	D
pB66_A-156	5p/3p	Mus musculus - GenMatch GID: 60459020	-1..526	IF		100.0	88.9	D
pB66_A-68	5p/3p	Mus musculus - GenMatch GID: 28881814	-1..430	IF		98.6	99.5	D
pB66_A-77	5p/3p	Mus musculus - GenMatch GID: 57012521	-1..697	IF		99.7	100.0	D
pB66_A-79	5p/3p	Mus musculus - GenMatch GID: 51315686	-1..516	IF		94.4	100.0	D
pB66_A-109	5p/3p	Mus musculus - GenMatch GID: 33285224	-1..1005	IF		99.8	100.0	D
pB66_A-175	5p/3p	Mus musculus - GenMatch GID: 33285224	-1..1005	IF		99.5	98.8	D
pB66_A-233	5p/3p	Mus musculus - GenMatch GID: 33285224	-1..1005	IF		99.3	99.4	D
pB66_A-136	5p/3p	Mus musculus - GenMatch GID: 33285224	-1..1005	IF		99.9	99.7	D
pB66_A-124	5p/3p	Mus musculus - GenMatch GID: 33285224	-1..1005	IF		100.0	99.5	D
pB66_A-189	5p/3p	Mus musculus - GenMatch GID: 12642989	-1	IF		100.0	60.6	D
pB66_A-145	5p/3p	Mus musculus - GenMatch GID: 24527497	-1..383	IF		99.2	97.4	D
pB66_B-2	5p/3p	Mus musculus - GenMatch GID: 42475643	-1..405	IF		99.8	100.0	D
pB66_A-14	5p/3p	Mus musculus - GenMatch GID: 28300643	-1..313	IF		100.0	100.0	D
pB66_A-199	5p/3p	Mus musculus - GenMatch GID: 34787446	-1..509	IF		100.0	89.8	D
pB66_B-57	5p/3p	Mus musculus - GenMatch GID: 21322392	-1..501	IF		98.4	100.0	D
pB66_A-244	5p/3p	Mus musculus - GenMatch GID: 24459826	-1..393	IF		100.0	100.0	D
pB66_A-193	5p/3p	Mus musculus - GenMatch GID: 24459826	-1..393	IF		100.0	99.5	D
pB66_B-59	5p/3p	Mus musculus - GenMatch GID: 24459826	-1..393	IF		100.0	99.7	D
pB66_A-163	5p/3p	Mus musculus - GenMatch GID: 24459826	-1..393	IF		100.0	100.0	D
pB66_A-23	5p/3p	Mus musculus - GenMatch GID: 24459826	-1..393	IF		100.0	100.0	D

Clone Name	Type Seq	Gene Name (Best Match)	Start..Stop (nt)	Frame	Sens	%Id 5p	%Id 3p	PBS
pB66_A-170	5p/3p	Mus musculus - GenMatch GID: 24459826	-1..393	IF		100.0	100.0	D
pB66_A-144	5p/3p	Mus musculus - GenMatch GID: 24459826	-1..393	IF		100.0	100.0	D
pB66_A-192	5p/3p	Mus musculus - GenMatch GID: 24459826	-1..393	IF		100.0	100.0	D
pB66_A-95	5p/3p	Mus musculus - GenMatch GID: 24459826	-1..393	IF		100.0	100.0	D
pB66_A-19	5p/3p	Mus musculus - GenMatch GID: 24459826	-1..393	IF		100.0	100.0	D
pB66_A-2	5p/3p	Mus musculus - GenMatch GID: 24459826	-1..393	IF		100.0	100.0	D
pB66_A-264	5p/3p	Mus musculus - GenMatch GID: 81295619	-1	IF		ALU 92.5	93.8	D
pB66_A-271	5p	Mus musculus - GenMatch GID: 34787446	-1	IF		100.0		D
pB66_A-274	5p/3p	Mus musculus - GenMatch GID: 49035075	-1..780	IF		100.0	95.1	D
pB66_A-147	5p/3p	Mus musculus - GenMatch GID: 74039307	-1..682	IF		99.4	99.7	D
pB66_B-27	3p	Mus musculus - GenMatch GID: 21065354	..666	??			100.0	N/A
pB66_A-246	3p	Mus musculus - GenMatch GID: 47078043	-1..463	IF			100.0	D
pB66_A-220	5p/3p	Mus musculus - GenMatch GID: 47078043	-1..463	IF		97.4	99.8	D
pB66_A-58	5p/3p	Mus musculus - GenMatch GID: 47078043	-1..463	IF		100.0	100.0	D
pB66_A-67	5p/3p	Mus musculus - GenMatch GID: 47078043	-1..463	IF		100.0	100.0	D
pB66_A-224	5p/3p	Mus musculus - GenMatch GID: 47078043	-1..463	IF		100.0	100.0	D
pB66_A-44	5p/3p	Mus musculus - GenMatch GID: 47078043	-1..463	IF		96.6	100.0	D
pB66_B-65	3p	Mus musculus - GenMatch GID: 47078043	..539	??			99.0	N/A
pB66_A-275	5p	Mus musculus - GenMatch GID: 58652300	-1	IF		100.0		D
pB66_B-63	5p/3p	Mus musculus - GenMatch GID: 62123204	-1..1255	IF		100.0	93.6	C
pB66_A-81	5p/3p	Mus musculus - GenMatch GID: 62123204	9..424	IF		100.0	97.1	C
pB66_A-130	5p/3p	Mus musculus - GenMatch GID: 62123204	9..424	IF		100.0	99.5	C
pB66_B-13	5p/3p	Mus musculus - GenMatch GID: 34495144	-1	IF		98.5	83.3	D
pB66_A-133	5p/3p	Mus musculus - GenMatch GID: 34495144	-1	IF		99.6	ALU 83.3	D
pB66_A-82	5p/3p	Mus musculus - GenMatch GID: 34495144	-1	IF		98.6	83.3	D
pB66_B-49	5p/3p	Mus musculus - GenMatch GID: 33438668	-1..589	OOF1		100.0	86.6	N/A
pB66_B-48	5p/3p	Mus musculus - GenMatch GID: 21671875	-1..744	IF		98.1	98.6	D
pB66_B-15	5p/3p	Mus musculus - GenMatch GID: 21671875	-1..744	IF		97.7	99.2	D
pB66_A-48	5p/3p	Mus musculus - GenMatch GID: 21671875	-1..744	IF		97.2	99.2	D
pB66_A-240	5p/3p	Mus musculus - GenMatch GID: 21671875	-1..738	IF		100.0	82.2	D

pB66_A-186	5p/3p	Mus musculus - GenMatch GID: 26006611	-1..511	IF		100.0	100.0	D
pB66_A-153	5p/3p	Mus musculus - GenMatch GID: 26006611	-1..511	IF		99.8	99.8	D
pB66_B-31	5p/3p	Mus musculus - GenMatch GID: 37719149	-1..343	IF		100.0	82.6	D
pB66_B-92	5p/3p	Mus musculus - GenMatch GID: 30985141	-1..489	IF		100.0	95.5	D
pB66_A-100	5p/3p	Mus musculus - GenMatch GID: 30985141	-1..490	IF		100.0	87.6	D
pB66_A-121	5p/3p	Mus musculus - GenMatch GID: 68270833	-1..386	IF		100.0	100.0	D
pB66_B-81	5p/3p	Mus musculus - GenMatch GID: 82465620	-1..565	IF		100.0	60.6	D
pB66_A-20	5p/3p	Mus musculus - GenMatch GID: 66773599	-1..783	IF		99.7	99.8	D
pB66_B-68	5p/3p	Mus musculus - GenMatch GID: 76362883	-1..571	IF		100.0	93.2	D
pB66_B-67	5p/3p	Mus musculus - GenMatch GID: 33438668	-1..624	OOF1		98.7	93.1	N/A
pB66_A-277	5p/3p	Mus musculus - GenMatch GID: 50839068	-1	IF		96.1	78.6	D
pB66_B-32	5p/3p	Mus musculus - GenMatch GID: 50839068	-1	IF		98.7	77.8	D
pB66_A-123	5p/3p	Mus musculus - GenMatch GID: 50839068	-1	IF		99.9	68.2	D
pB66_A-129	5p/3p	Mus musculus - GenMatch GID: 50839068	-1	IF		99.8	68.2	D
pB66_B-14	5p/3p	Mus musculus - GenMatch GID: 81778322	-1..1165	OOF1		100.0	93.6	N/A
pB66_A-111	5p/3p	Mus musculus - GenMatch GID: 81778246	-1..517	IF		98.5	99.8	D
pB66_A-66	5p/3p	Mus musculus - GenMatch GID: 60459020	-1..533	IF		99.8	100.0	D
pB66_A-62	5p/3p	Mus musculus - GenMatch GID: 60459020	-1..533	IF		100.0	100.0	D
pB66_B-9	3p	Mus musculus - GenMatch GID: 60459020	..1047	??			100.0	D
pB66_A-197	3p	Mus musculus - GenMatch GID: 60459020	..490	??			99.2	D
pB66_A-219	5p/3p	Mus musculus - GenMatch GID: 76683163	-1..323	IF		99.4	94.1	D
pB66_A-206	5p/3p	Mus musculus - GenMatch GID: 76683163	-1..323	IF		98.5	94.1	D
pB66_A-204	5p/3p	Mus musculus - GenMatch GID: 76683163	-1..323	IF		98.8	94.4	D
pB66_A-36	5p/3p	Mus musculus - GenMatch GID: 81295619	-1	IF		ALU 92.9	93.8	D
pB66_A-141	5p/3p	Mus musculus - GenMatch GID: 84794837	-1..314	IF		100.0	100.0	D
pB66_A-6	5p/3p	Mus musculus - GenMatch GID: 84794837	-1..314	IF		100.0	100.0	D
pB66_A-39	3p	Mus musculus - GenMatch GID: 34495144	..645	??			97.5	N/A
pB66_A-113	5p/3p	Mus musculus - GenMatch GID: 83313863	-1	IF		100.0	64.7	D
pB66_A-200	5p/3p	Mus musculus - GenMatch GID: 83313863	-1	IF		99.7	64.7	D
pB66_A-43	5p/3p	Mus musculus - GenMatch GID: 83313863	-1	IF		100.0	64.7	D

pB66_A-72	5p/3p	Mus musculus - GenMatch GID: 83313863	-1	IF		99.8	64.7	D
pB66_A-230	5p/3p	Mus musculus - GenMatch GID: 81295619	-1	IF		100.0	65.1	D
pB66_B-50	5p/3p	Mus musculus - GenMatch GID: 81295619	-1	IF		ALU 92.8	93.8	D
pB66_A-63	5p/3p	Mus musculus - GenMatch GID: 45825198	-1..609 ✗	IF		100.0	ALU 60.2	D
