# Supplementary material for: Identification of novel human microcephaly-linked protein Mtss2 that mediates cortical progenitor cell division and corticogenesis through Nedd9-RhoA
Source: eLife. 2025 Jul 23;13:RP92748. doi: 10.7554/eLife.92748 (PMC12286603; doi:10.7554/eLife.92748)
Supplement: Supplementary file 2. [file elife-92748-supp2.pdf]

Table 1

Variant	c.2011C>T, heterozygous
	p.R671W
	de novo
Current age	15 years old
Gender	M
Family History	Caucasian, dutch
	27
Growth	(- 2SD)
Microcephaly	Borderline microcephaly
Neurological examination	yes
Intellectual disability	Yes, mild ID IQ69
Seizures	No
Behavioral problems	Autism spectrum disorder, ADHD
Ophthalmological anomalies	Congenital nystagmus
Craniofacial dysmorphisms	Small ears with abnormal hlices, ptosis left, epicanthus, diastasis teeth,
Urorenal abnormalities	No
Skeletal abnormalities	No
